# PPIB and its role in prostate cancer: associations with transcriptomic and splicing alterations

**DOI:** 10.3389/fonc.2026.1864404

**Published:** 2026-09-08

**Authors:** Shuangyu Ma, Aikeshanjiang Ailiyaer, Huixiang Chen, Yishen Mao, Mulati Rexaiti, Abdureheman Zebibula

**Affiliations:** Department of Urology, First Affiliated Hospital of Xinjiang Medical University, Urumqi, China

**Keywords:** alternative splicing, PPIB, prostate cancer, RNA-Seq, transcriptomics

## Abstract

**Background:**

While peptidyl-prolyl cis-trans isomerase B (PPIB) has been implicated in tumorigenesis and RNA metabolism, its specific function in prostate cancer is poorly understood.

**Methods:**

PPIB expression was assessed in clinical prostate adenocarcinoma samples and the TCGA-PRAD cohort. In PC-3 cells, stable PPIB knockdown was achieved via lentiviral shRNA. Subsequent *in vitro* assays measured proliferation, apoptosis, migration, and invasion, and tumor growth was evaluated in nude mouse xenograft models. We conducted strand-specific RNA sequencing to identify differentially expressed genes (DEGs) and PPIB-associated alternative splicing events (ASEs), followed by functional enrichment analyses (GO, KEGG, and GSEA). In addition, an shRNA-resistant PPIB cDNA carrying synonymous substitutions within the sh-PPIB-275 target sequence was re-expressed in PPIB-depleted cells, followed by Transwell rescue assays.

**Results:**

TCGA-PRAD analysis showed significantly higher PPIB mRNA expression in tumors. In the paired IHC cohort, an exploratory ROI-level comparison showed higher PPIB H-scores in tumor regions than in adjacent non-tumor regions (P < 0.05). Because multiple ROIs were obtained from the same patients, patient-level inference was based on ROI-averaged paired values; the mean tumor H-score was higher, but the paired difference was not statistically significant. PPIB knockdown inhibited cell proliferation, migration, invasion, and xenograft tumor growth. A modest increase in apoptotic cells was observed after PPIB depletion. Transcriptomic analysis revealed 570 differentially expressed genes and 873 altered splicing events, with enriched pathways involving DNA repair, the cell cycle, and amino acid metabolism. Integrating DEG and ASE data identified 22 overlapping genes, including the metabolic regulators PHGDH and SHMT2. Re-expression of shRNA-resistant PPIB significantly reversed the reductions in migration and invasion caused by PPIB knockdown.

**Conclusions:**

PPIB promotes prostate cancer progression and is associated with broad transcriptional and alternative splicing alterations. These results indicate a role for PPIB in prostate cancer progression and establish a foundation for investigating its post-transcriptional functions.

## Introduction

Prostate cancer ranks among the most frequently diagnosed malignancies in men worldwide and continues to be a major contributor to cancer-associated mortality. Well-documented risk determinants encompass older age, African descent, inherited pathogenic mutations in DNA repair and susceptibility genes—including *BRCA2*, *BRCA1*, *ATM*, *CHEK2*, and *HOXB13*— along with a positive family history of prostate cancer (1). Lifestyle and environmental factors, including diet, obesity, physical inactivity, chronic inflammation, metabolic dysfunction, recurrent genitourinary infections, and exposure to endocrine-disrupting chemicals or ionizing radiation, also contribute to disease risk (2).

Prostate cancer arises through the combined effects of inherited genetic risk, epigenetic remodeling, and environmental exposure. Germline pathogenic variants in DNA repair genes, including *BRCA2*, *BRCA1*, *ATM*, *CHEK2*, and *HOXB13*, contribute to familial clustering and early-onset disease (3). Meanwhile, persistent inflammation, aberrant androgen receptor activity, and dysregulation of signaling pathways, such as PI3K/AKT/mTOR, RAS/RAF/MEK/ERK, and Wnt/β-catenin, promote tumor growth, invasion, and treatment resistance (4). Epigenetic dysregulation—encompassing aberrant DNA methylation, altered histone marks, and disrupted noncoding RNA expression—plays a key role in driving prostate cancer progression (5).

Aberrant RNA processing has become a recognized feature of cancer progression, and alternative splicing is increasingly implicated in prostate cancer biology. Splicing alterations can affect genes involved in proliferation, apoptosis, metabolism, invasion, and therapy response. *PPIB*, a cyclophilin family member, is best known for its peptidyl-prolyl isomerase activity, but emerging evidence suggests a possible role in RNA-related regulation (6, 7). In prostate cancer, however, the expression pattern, functional role, and relationship of *PPIB* to transcriptomic and splicing changes remain unclear. We therefore examined *PPIB* expression and function using clinical specimens, cell-based assays, and xenograft models, and performed a transcriptome analysis to identify downstream pathways associated with *PPIB* depletion.

## Materials and methods

### Lentivirus information

The shRNA sequence targeting PPIB (5′-CTACAGGAGAGAAAGGATTTG-3′), hereafter referred to as sh-PPIB-275, and the negative control sequence (5′-TTCTCCGAACGTGTCACGT-3′) were cloned into the LV-3 (pGLVH1/GFP+Puro) lentiviral vector. All viral particles were provided by GenePharma (Suzhou, China) for subsequent cell infection.

### Immunohistochemistry

Immunohistochemistry (IHC) was carried out on paired formalin-fixed, paraffin-embedded (FFPE) specimens from 15 patients with histopathologically verified prostate carcinoma. For each case, prostate cancer tissue and matched adjacent benign prostate tissue were analyzed side by side to facilitate a within-subject comparative assessment. For quantitative image analysis, three representative regions of interest (ROIs) were evaluated per tissue sample when available; the current analyzable dataset included 45 tumor ROIs and 44 adjacent non-tumor ROIs. Following standard deparaffinization and rehydration through descending ethanol concentrations, heat-induced antigen retrieval was performed using either EDTA (pH 9.0) or citrate (pH 6.0) buffer, selected based on antibody-specific validation data. Endogenous peroxidase activity was blocked by incubating the tissue in 3% hydrogen peroxide, followed by serum blocking to reduce nonspecific antibody binding. Primary antibodies were applied overnight at 4°C; detection used HRP-conjugated secondary antibodies incubated at 37°C for 1 h. Signal development employed 3,3′-diaminobenzidine (DAB), followed by hematoxylin counterstaining, graded alcohol dehydration, xylene clearing, and permanent mounting. Whole-slide images were digitized and processed using QuPath v4.3, OpenSlide, and custom Python scripts. Tissue regions were annotated with pathology-assisted digital image analysis, and total cells, positive cells, positive rate, and H-score were calculated for each ROI. The H-score was calculated as 1 × (% cells with 1+ staining) + 2 × (% cells with 2+ staining) + 3 × (% cells with 3+ staining), where 1+, 2+, and 3+ represent weak, moderate, and strong staining intensity, respectively. All automated outputs underwent pathology review to verify segmentation fidelity and scoring accuracy; formal blinding to tissue group or clinicopathological variables was not documented.

The available clinicopathological characteristics of the IHC cohort, including age, preoperative PSA level, histological diagnosis, Gleason score, ISUP grade group, and pathological stage, are summarized in **Table 1**.

**Table 1:** Clinicopathological and IHC characteristics of the paired tissue cohort.

Variable	Summary/distribution
Included cases	15 paired prostate cancer and adjacent non-tumor FFPE tissue cases
Age, years	67.9 ± 5.5; range 59–77
Preoperative PSA, ng/mL	Numeric values: n = 13, median 12.40, range 1.17–67.00; >100: n = 1; NA: n = 1
Histological type	Acinar adenocarcinoma of the prostate: 15
Gleason score	4 + 5 = 9: 1; 4 + 4 = 8: 3; 3 + 3 = 6: 2; 4 + 3 = 7: 3; 3 + 4 = 7: 4; 3 + 5 = 8: 2
ISUP grade group	5: 1; 4: 5; 1: 2; 3: 3; 2: 4
pT stage	pT3a: 6; pT2a: 3; pT2c: 4; pT3b: 2
pN stage	pN0: 15
pM stage	pM0: 14; pM1: 1
ROI structure	45 tumor ROIs and 44 adjacent non-tumor ROIs
IHC quantification	H-scores were quantified by ROI-level digital image analysis. The ROI-level comparison was exploratory; primary inference used paired patient-level means after averaging ROIs within each tissue sample.
Blinding	Formal blinding was not documented

FFPE, formalin-fixed, paraffin-embedded; IHC, immunohistochemistry; ISUP, International Society of Urological Pathology; NA, not available; PSA, prostate-specific antigen; ROI, region of interest.

### Cell culture and lentiviral transduction

PC-3 cells (catalog no. ZQ0040; Zhongqiao Xinzhou Biotechnology, China) were propagated in RPMI-1640 medium supplemented with 10% heat-inactivated fetal bovine serum (Gibco, #10091148), 100 U/mL penicillin, and 100 µg/mL streptomycin (HyClone, #SV30010), under standard conditions: 37°C in a humidified atmosphere containing 5% CO_2_. Lentiviral particles were delivered at an MOI of 25 to ensure efficient transduction. Forty-eight hours post-transduction, selection with 0.3 µg/mL puromycin was initiated to isolate stably transfected clones. PPIB knockdown was evaluated at the transcript level by qRT-PCR and confirmed at the protein level by Western blotting.

### shRNA-resistant PPIB rescue and phenotypic assays

To assess the specificity of the phenotypes associated with PPIB depletion, Flag-tagged PPIB cDNA containing synonymous substitutions within the sh-PPIB-275 target sequence was re-expressed in PPIB-depleted PC-3 cells, rendering the ectopic transcript resistant to sh-PPIB-275 without altering the encoded amino acid sequence. Three groups were analyzed: PC-3-sh-NC + OE-NC, PC-3-sh-PPIB-275 + OE-NC, and PC-3-sh-PPIB-275 + OE-PPIB. PPIB re-expression was verified by qRT-PCR and anti-Flag Western blotting, and migration and invasion were evaluated using Transwell assays. Three independent biological experiments were performed for each group.

### Assessment of gene expression

First-strand cDNA synthesis was carried out from total RNA using the Vazyme HiScript III RT SuperMix (R323-01), following the supplier’s protocol. Quantitative reverse transcription PCR (qRT-PCR) was conducted on an ABI QuantStudio 5 Real-Time PCR System using the following thermal profile: 95°C for 10 min, followed by 40 cycles of denaturation at 95°C for 15 s and annealing/extension at 60°C for 60 s. Each sample was analyzed in triplicate to ensure technical reproducibility. GAPDH served as the endogenous reference gene, and relative transcript levels were determined using the 2−ΔΔCt method (8). Because formal stability validation of GAPDH was not performed under the present experimental conditions, GAPDH-normalized qRT-PCR results were interpreted as supportive evidence and considered together with protein-level validation and sequencing-based analyses. Primer sequences are provided in **Supplementary Table 1**. Representative DEGs were assessed by qRT-PCR, whereas selected alternative splicing events were evaluated separately using event-specific primer sets. Three independent biological replicates were analyzed per group, and each biological sample was measured in three technical replicates. Gene expression statistics were based on sample-level ΔCt values, and AS ratios were calculated for each biological replicate before group comparison.

### Western blotting

Total cellular proteins were extracted from PC-3 cells using ice-cold RIPA lysis buffer (Proteintech, #PR20001), supplemented with a comprehensive protease inhibitor mixture (Sigma, #4693116001). Following 30 min of incubation on ice, the lysates were centrifuged at 12,000 × g for 15 min at 4°C to pellet insoluble debris; the resulting supernatant was collected as the whole-protein extract. Protein concentrations were determined using a standardized assay, and equalized amounts were denatured by boiling in Laemmli sample buffer (Solarbio, #P1040) for 10 min. The denatured proteins were resolved via SDS-PAGE and electrophoretically transferred onto 0.45-µm PVDF membranes (Millipore). The membranes were blocked for 1 h at room temperature in TBST containing 5% nonfat dry milk. Primary antibodies targeting PPIB (1:1,000; Proteintech, #11607-1-AP) and β-actin (1:4,000; Proteintech, #20536-1-AP) were applied overnight at 4°C. Additional immunoblotting assessed cleaved caspase-3, cleaved PARP1, N-cadherin, E-cadherin, Vimentin, SHMT2, GINS1, and PHGDH, with β-actin as the loading control. After washing, the membranes were incubated with the appropriate HRP-conjugated secondary antibody for 45 min at room temperature. Immunoreactive bands were detected by enhanced chemiluminescence, and densitometric values were normalized to β-actin.

### Cell proliferation assay

Cell proliferation was indirectly assessed by measuring metabolic activity via the Cell Counting Kit-8 (CCK-8; MedChemExpress, HY-K0301). PC-3 cells were seeded at a density of 1 × 10^4^ cells/well in 24-well culture plates and incubated under standard conditions (37°C, 5% CO_2_, humidified atmosphere) for 24 h to ensure attachment and stabilization. Subsequently, 10 µL of CCK-8 reagent was added to each well in both the PPIB knockdown (sh-PPIB) and non-targeting control (sh-NC) groups. Parallel blank wells—containing medium only, without cells—were included to account for background signal. After a 3-h incubation at 37°C, absorbance was measured at 450 nm using a BioTek ELX800 microplate reader. Proliferation levels were expressed as normalized optical density values, calculated by subtracting the mean blank absorbance from the raw OD readings of each treatment group.

### Flow cytometric analysis of cell apoptosis

Apoptosis was measured with an Annexin V/PI apoptosis detection kit (KeyGEN Biotech, China; #40304ES60). PC-3 cells were seeded in 24-well plates at a density of 1 × 10_5_ cells per well and transduced with the appropriate shRNA constructs. After treatment, the cells were collected and stained with 5 µL of Annexin V-Fluor 647 and 10 µL of propidium iodide (PI) for 15 min at room temperature in the dark. The proportion of apoptotic cells was then analyzed using a FACSCanto flow cytometer (BD Biosciences, USA).

### Cell migration assay

Cell migration was quantified using Corning Transwell permeable supports with 8-µm pore membranes. PC-3 cells (1 × 10_5_ cells per insert) were resuspended in serum-free RPMI-1640 medium and seeded into the upper chamber, while the lower chamber was filled with complete medium supplemented with 10% fetal bovine serum (FBS; Gibco, #10091148) to serve as a chemoattractant. Following 48 h of incubation at 37°C in a humidified 5% CO_2_ atmosphere, the non-migrated cells on the apical side of the membrane were carefully removed with sterile cotton-tipped applicators. The migrated cells that had adhered to the basolateral surface were fixed with 4% paraformaldehyde for 20 min at room temperature, stained with 0.1% crystal violet for 15 min, and air-dried. Images were acquired using an inverted microscope (Mshot MF52-N) at 200× magnification, and migrated cells were manually counted across five randomly selected fields per insert.

### Cell invasion assay

Invasive capacity was evaluated using Corning BioCoat Matrigel Invasion Chambers (8-µm pore size; #3422). Before cell seeding, the apical surface of each insert was uniformly coated with 100 µL of ice-cold Matrigel (BD Biosciences), diluted 1:8 in serum-free medium, and allowed to polymerize at 37°C for 1 h. A total of 1.5 × 10_5_ PC-3 cells suspended in 200 µL serum-free medium were seeded into the upper chamber, while 600 µL of complete medium supplemented with 10% fetal bovine serum (FBS; Gibco, #10091148) was placed in the lower chamber to establish a chemotactic gradient. Following 48 h of incubation under standard culture conditions (37°C, 5% CO_2_, humidified atmosphere), the non-invading cells on the apical side were carefully removed using sterile cotton-tipped applicators. The cells that had traversed the Matrigel barrier and adhered to the basolateral surface were fixed with 4% paraformaldehyde for 20 min at room temperature, stained with 0.1% crystal violet for 15 min, and air-dried. Images were acquired at 200× magnification using an inverted microscope (Mshot MF52-N), and the number of invasive cells was quantified by manual enumeration across five randomly selected fields per insert.

### Tumor Xenografts in nude mice

A total of 10 nude mice were randomly divided into the sh-NC and sh-PPIB-275 groups (n = 5 per group). The mice were monitored during the experiment, and the monitoring frequency was adjusted according to tumor growth. Tumor size and body weight were recorded, and tumor size was measured on days 4, 5, 6, 7, 8, 11, and 12 after inoculation. All mice were euthanized on day 13 for tumor collection. Mice were euthanized earlier if the longest tumor diameter approached 15 mm, or if they showed marked distress, impaired mobility, ulceration, or sustained body weight loss of more than 10%. No ulceration was observed, body weight loss remained within 10%, and no mice died before the endpoint.

For surgery, anesthesia was induced via an intraperitoneal injection of 2.5% tribromoethanol (10 µL/g). For examinations, isoflurane inhalation anesthesia was used (induction 3–5%, maintenance 1–2%, oxygen flow 1 L/min). Euthanasia was performed by intraperitoneal injection of 2.5% tribromoethanol (50 µL/g).

### RNA extraction and sequencing

Total RNA was extracted and subsequently subjected to on-column DNase I digestion (RQ1 RNase-Free DNase; Promega) to eliminate residual genomic DNA. The RNA concentration and purity were assessed by measuring the A260/A280 ratio using a Bio-Rad SmartSpec Plus spectrophotometer, and RNA integrity was evaluated by 1.5% agarose gel electrophoresis. A total of 1 µg of high-quality RNA was used for library construction, and an additional DNase treatment was performed before reverse transcription. Strand-specific RNA-seq libraries were generated using the VAHTS^®^ Universal V8 RNA-seq Library Prep Kit (Vazyme, catalog #N605) in accordance with the vendor’s recommended protocol. Poly(A)+ mRNA was enriched using VAHTS mRNA Capture Beads (Vazyme, #N401), followed by fragmentation. First- and second-strand cDNA were synthesized, with dUTP incorporated during second-strand synthesis to preserve strand information. The resulting cDNA fragments were end-repaired, A-tailed, and ligated to VAHTS RNA Multiplex Oligos (Vazyme, #N323). During PCR amplification, the dUTP-containing strand was not amplified, thereby preserving strand specificity. The purified libraries were quantified with the Qubit fluorometric assay, size-distributed using an Agilent Bioanalyzer 2100 system, and archived at −80°C. High-throughput sequencing was carried out on the Illumina NovaSeq X Plus platform with paired-end 2 × 150 bp reads.

### RNA-seq read cleaning and alignment

Raw RNA-seq reads underwent rigorous quality assessment before further analysis. Low-quality sequences, including those with ≥2 ambiguous bases (N), adapter contamination, Phred scores <20, or length <16 nt, were filtered out using the HO-FASTX Toolkit (v3.0.13). High-quality reads were aligned to the GRCh38.p14 human reference genome with HISAT2 (v2.2.1), permitting up to four base mismatches per alignment (9). Transcript abundance at the gene level was calculated from uniquely aligned sequencing reads and normalized to FPKM (fragments per kilobase of transcript per million mapped reads).

Transcript reconstruction was performed with StringTie (v2.1.6). Differential gene expression was assessed independently using DESeq2 (v1.38.3) and edgeR (v3.40.2) in R (v4.3.3). Functional annotation and pathway enrichment were performed using KOBAS 2.0. MultiQC v1.14 was employed to aggregate and visualize key sequencing quality metrics, including base-call accuracy, PCR duplication levels, mapping efficiency, and transcriptome coverage evenness.

### Differential gene expression analysis

Differential expression analysis was performed with DESeq2 (v1.38.3) in the R/Bioconductor framework. Genes with an adjusted *p-value of* ≤ 0.05 and an absolute fold change of at least two (fold change ≥ 2.0 or ≤ 0.5) were classified as differentially expressed (10). Raw read counts were used for statistical testing, whereas FPKM and TPM values were only used for graphical display.

### Alternative splicing analysis

Regulated alternative splicing events (RASEs) were detected and quantified using the ABLas bioinformatics pipeline (Ablife, Wuhan, China). Based on splice-junction read alignments, ABLas identified six major event types: exon skipping (ES), intron retention (IR), alternative 5′ splice-site usage (A5SS), alternative 3′ splice-site selection (A3SS), mutually exclusive exons (MXE), and multiexon skipping (MES). An event was considered differentially regulated when it met all three criteria: (i) an absolute difference in percent spliced in (ΔPSI) of ≥ 0.15, (ii) a false discovery rate (FDR) of < 0.05, and (iii) at least 10 supporting junction-spanning reads, excluding intronic-only reads.

### Functional enrichment analysis

Functional enrichment analysis of differentially expressed genes (DEGs) was performed with KOBAS 2.0 to detect significantly overrepresented Gene Ontology (GO) terms and Kyoto Encyclopedia of Genes and Genomes (KEGG) pathways (11). Statistical significance was assessed using the hypergeometric test, and *p-*values were adjusted for multiple testing using the Benjamini–Hochberg false discovery rate (FDR) method. Results were considered significant only when the FDR-corrected q-value was below 0.05.

### Gene set enrichment analysis

To investigate the biological pathways perturbed by *PPIB* knockdown, gene set enrichment analysis (GSEA) was conducted using the clusterProfiler package in R (12). Genes were ranked based on their log_2_ fold-change values when comparing the sh-PPIB group to the control (sh-NC). This ranked list was subsequently tested for enrichment against the MSigDB Hallmark collection—a curated set of well-defined, pathway-centric gene signatures—to detect coherent transcriptional shifts at the pathway level (13).

### Statistical analysis

All statistical analyses and graphical representations were performed in R version 4.3.3. Continuous variables are presented as mean ± standard error of the mean (SEM) unless otherwise specified. For the paired IHC cohort, an exploratory ROI-level comparison was performed using all available ROI H-scores; this analysis did not account for within-patient clustering and was therefore not used as the primary inferential result. For the primary patient-level analysis, ROI-level H-scores were averaged within each tissue sample, and tumor and matched adjacent tissue means were compared using a two-tailed paired t-test. For pairwise comparisons between two independent groups, an unpaired two-sided t-test was applied; in cases where Levene’s test revealed significant heteroscedasticity (P < 0.05), Welch’s correction was implemented to account for unequal variances. For the three-group shRNA-resistant PPIB rescue experiments, an ordinary one-way analysis of variance followed by a Tukey’s multiple-comparison test was used. Rescue-assay data are presented as the mean ± SD, with each plotted point representing one independent biological experiment.

In the xenograft study, tumor volume trajectories were summarized descriptively using longitudinal growth curves. Formal hypothesis testing was restricted to endpoint measurements—namely, final tumor volume and weight. Statistical significance was defined as *P* < 0.05 (two-sided). All error bars denote SEM unless stated otherwise.

### Public dataset access

The TCGA-PRAD data used in this study were accessed for research purposes on 17 June 2025.

## Results

### PPIB expression in prostate cancer and the phenotypic effects of PPIB knockdown in PC-3 cells

**Figure 1:**
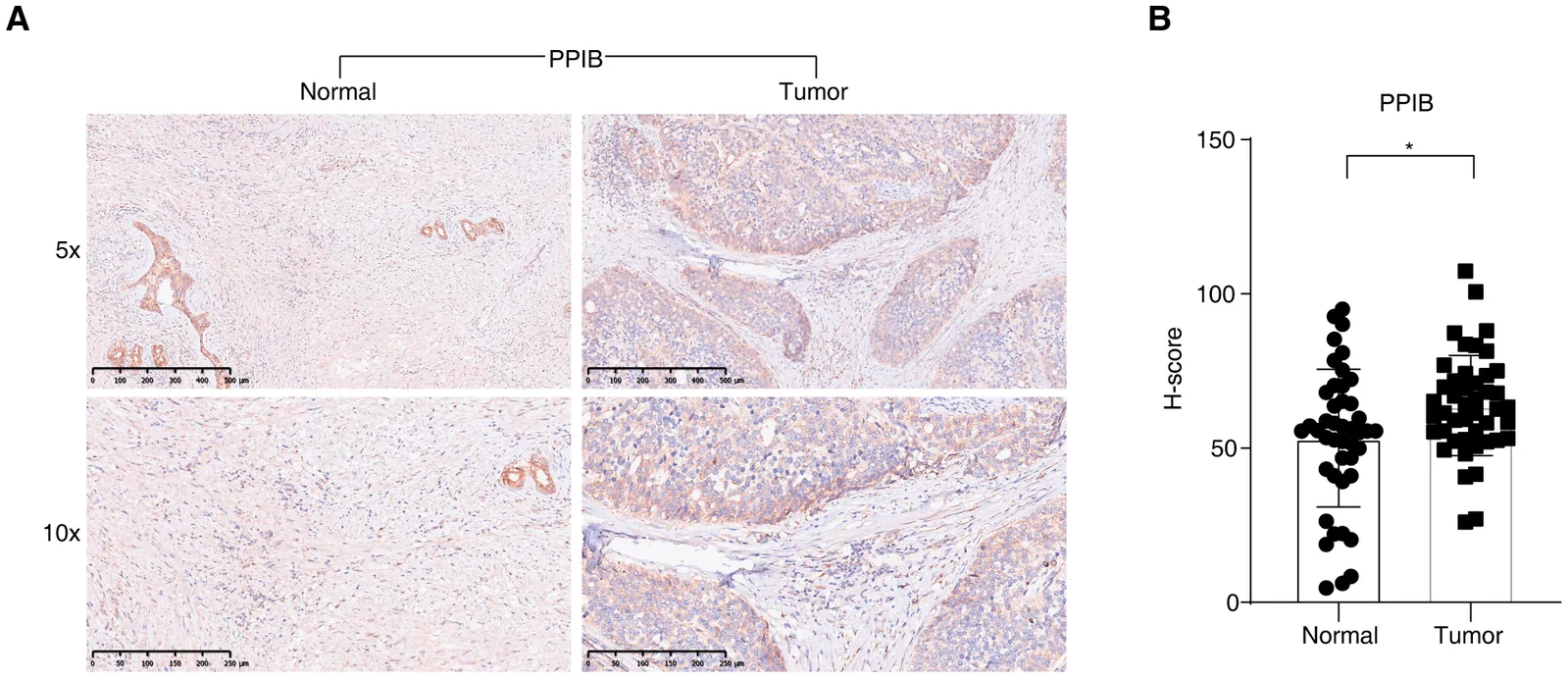
**(A)** Immunohistochemical staining of PPIB in prostate cancer tissues and matched adjacent non-tumor tissues. **(B)** Quantification of PPIB H-scores. Three ROIs were evaluated per tissue sample when available, yielding 45 tumor ROIs and 44 adjacent non-tumor ROIs across 15 paired cases. An exploratory ROI-level comparison showed higher H-scores in tumor regions (P < 0.05), but this comparison did not account for within-patient clustering. For the primary patient-level analysis, ROI values were averaged within each tissue sample. The mean tumor H-score was higher than the mean H-score of the matched adjacent tissue (63.76 ± 11.10 vs 52.59 ± 20.79), but the paired difference was not statistically significant (two-tailed paired t-test, P = 0.0738; n = 15 paired cases). *P < 0.05.

**Figure 2:**
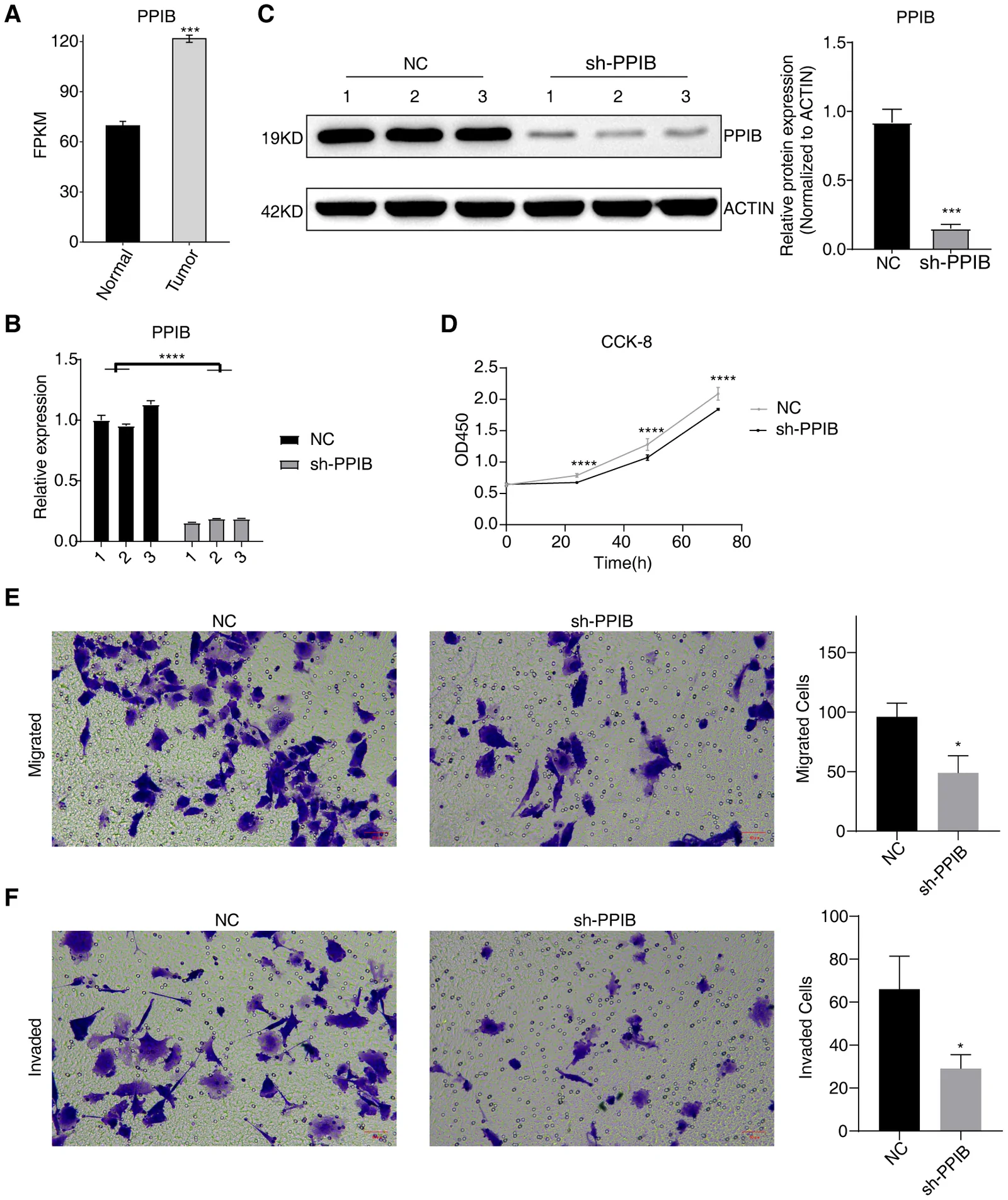
**(A)** PPIB expression in prostate cancer based on TCGA-PRAD data. **(B)** qRT-PCR analysis of PPIB mRNA in PPIB-knockdown PC-3 cells. ****P < 0.0001. **(C)** Western blot analysis of PPIB protein in PPIB-knockdown PC-3 cells. **(D)** Cell proliferation after PPIB knockdown. **(E)** Representative migration assay results after PPIB knockdown. **(F)** Representative invasion assay results after PPIB knockdown. Full-length blots are presented in **Supplementary Figure 2**. *P < 0.05, ***P < 0.001.

We first assessed PPIB expression in prostate cancer using 15 paired prostate cancer and adjacent non-tumor FFPE tissue specimens. For quantitative IHC analysis, three ROIs were evaluated per tissue sample, when available, resulting in 45 tumor ROIs and 44 adjacent non-tumor ROIs. The clinicopathological characteristics of the 15 included cases are summarized in **Table 1**. PPIB staining was predominantly cytoplasmic (**Figure 1A**). An exploratory ROI-level comparison showed higher H-scores in tumor regions than in adjacent non-tumor regions (P < 0.05); however, this analysis treated multiple ROIs from the same patients as separate observations and did not account for within-patient clustering. Accordingly, the primary inferential analysis was performed after ROI values were averaged within each tissue sample. At the patient level, the mean tumor H-score was higher than the mean matched adjacent tissue H-score (63.76 ± 11.10 vs. 52.59 ± 20.79), but the paired difference was not statistically significant (two-tailed paired t-test, P = 0.0738; n = 15 paired cases) (**Figure 1B**). Analysis of the TCGA-PRAD cohort showed that PPIB mRNA expression was significantly higher in prostate adenocarcinoma tissues than in normal controls (**Figure 2A**). To investigate its functional role, we established a stable PPIB knockdown model in the androgen-independent prostate cancer cell line PC-3. qRT-PCR supported a reduction in PPIB transcript levels, and Western blotting confirmed corresponding protein depletion (**Figures 2B, C**). Functionally, PPIB depletion significantly inhibited PC-3 cell proliferation (**Figure 2D**). Flow cytometric analysis showed a modest increase in apoptotic cells in the sh-PPIB group compared to the sh-NC group (**Supplementary Figure 1**; two-tailed unpaired t-test, P < 0.05; n = 3). Given the low absolute apoptosis rate, this finding should be interpreted cautiously. PPIB knockdown also markedly reduced the migratory and invasive capacities of PC-3 cells, as shown by Transwell assays (**Figures 2E, F**).

Additional immunoblotting showed higher cleaved caspase-3 after PPIB knockdown, whereas cleaved PARP1 was not significantly changed. N-cadherin was reduced, while E-cadherin and Vimentin were not significantly altered; therefore, these markers did not show a coherent canonical EMT pattern. Total SHMT2 and PHGDH protein levels increased, whereas GINS1 decreased. These findings provide supportive protein-level evidence for selected expression changes, but they do not establish a causal mechanism or validate specific alternative splicing events (**Supplementary Figure 6**).

### shRNA-resistant PPIB re-expression reverses the impaired migration and invasion of PPIB-depleted PC-3 cells

**Figure 3:**
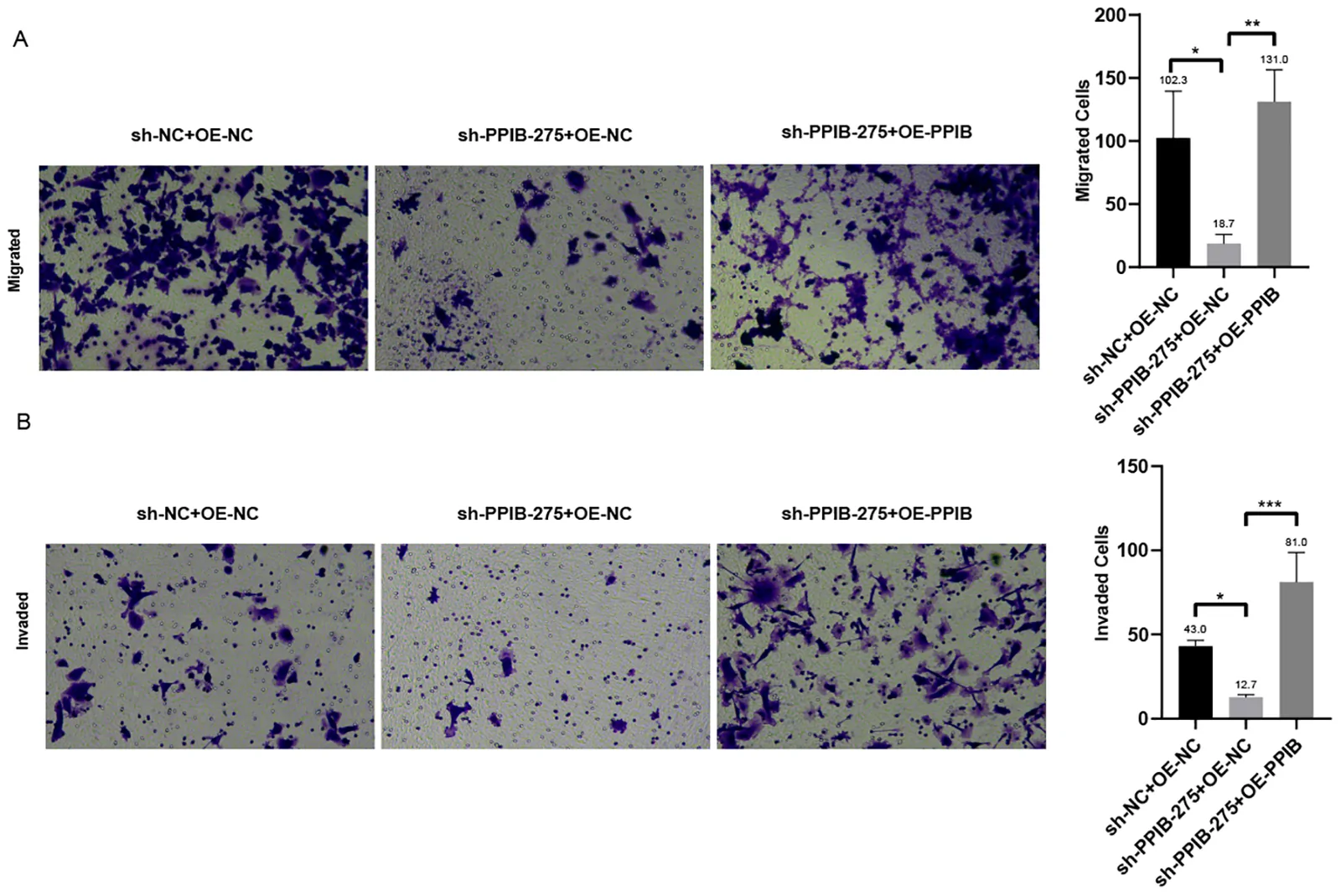
shRNA-resistant PPIB re-expression reverses the migration and invasion defects caused by PPIB knockdown. **(A)** Representative Transwell migration images and quantification. **(B)** Representative Matrigel invasion images and quantification for sh-NC + OE-NC, sh-PPIB-275 + OE-NC, and sh-PPIB-275 + OE-PPIB cells. Data are presented as the mean ± SD (n = 3 independent biological experiments). Statistical analysis was performed using an ordinary one-way ANOVA followed by a Tukey’s multiple-comparison test. *P < 0.05, **P < 0.01, ***P < 0.001.

To further assess the specificity of the migration and invasion phenotypes associated with PPIB depletion, Flag-tagged shRNA-resistant PPIB cDNA containing synonymous substitutions within the sh-PPIB-275 target sequence was re-expressed in sh-PPIB-275 PC-3 cells. Expression of the shRNA-resistant PPIB protein was confirmed by anti-Flag Western blotting and supported by qRT-PCR (**Supplementary Figure 5**). In the migration assay, the sh-PPIB-275 + OE-NC group showed significantly fewer migrated cells than the sh-NC + OE-NC group (Tukey-adjusted P = 0.0193), whereas shRNA-resistant PPIB re-expression significantly increased migration relative to the knockdown group (P = 0.0049) (**Figure 3A**). Migratory activity in the rescue group was not detectably different from that in the control group (P = 0.4326). Similarly, invasion was reduced by PPIB knockdown (P = 0.0280) and markedly increased after shRNA-resistant PPIB re-expression (P = 0.0005) (**Figure 3B**). The invasive activity of the rescue group exceeded that of the control group (P = 0.0103), potentially reflecting the high level of PPIB expression achieved by the rescue construct. These results support the specificity of the migration and invasion phenotypes associated with PPIB depletion, although the use of a single shRNA and a single cell line remains a limitation.

### PPIB knockdown is associated with reduced tumor growth in nude mice

**Figure 4:**
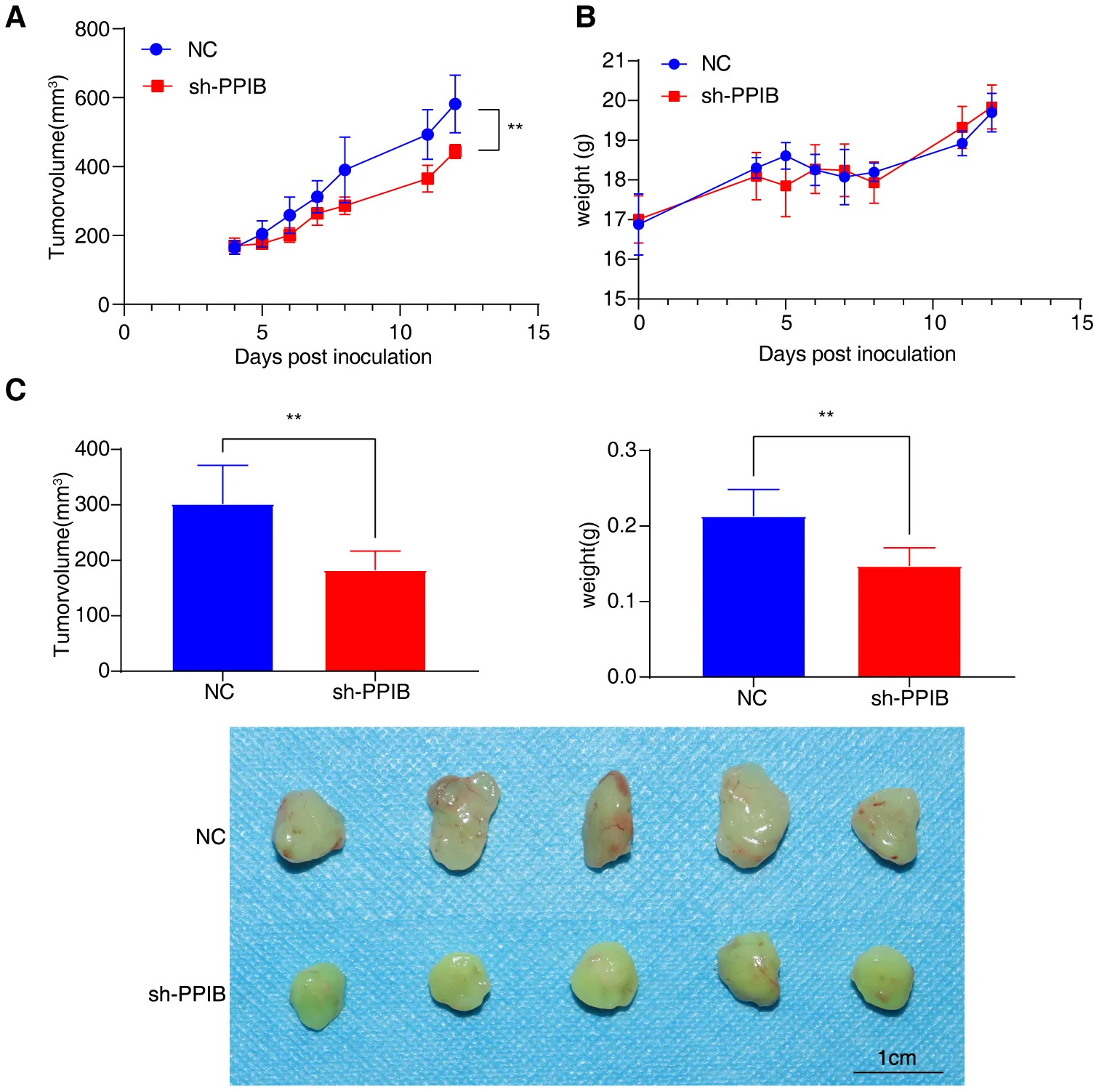
**(A)** Tumor growth curves in nude mice bearing PC-3-sh-NC or PC-3-sh-PPIB-275 xenografts (n = 5 per group). **(B)** Body weight curves during the experiment. **(C)** Tumor weight and tumor volume at harvest. Tumor weight: 0.147 g vs. 0.213 g, *P* = 0.0104; tumor volume: 182.4 mm³ vs. 301.8 mm³, *P* = 0.0146 (two-sided Welch’s t-test). **P < 0.01.

To evaluate the effect of *PPIB* knockdown on tumor growth *in vivo*, PC-3 cells stably expressing non-targeting shRNA (sh-NC) or PPIB-targeting shRNA (sh-PPIB-275) were subcutaneously implanted into nude mice (n = 5 per group). Tumor size was measured on days 4, 5, 6, 7, 8, 11, and 12 after implantation, and tumors were collected on day 13. Compared with the sh-NC group, xenografts derived from the sh-PPIB-275 cells grew more slowly and showed smaller tumor volumes during the observation period (**Figure 4A**). At the end of the experiment, both tumor weight and tumor volume were significantly lower in the sh-PPIB-275 group than those in the sh-NC group (tumor weight: 0.147 g vs. 0.213 g, *P* = 0.0104; tumor volume: 182.4 mm³ vs. 301.8 mm³, *P* = 0.0146; two-sided Welch’s t-test) (**Figure 4C**). Body weight remained comparable between the two groups throughout the experiment (**Figure 4B**).

### *PPIB* knockdown induces transcriptomic changes in PC-3 cells

**Figure 5:**
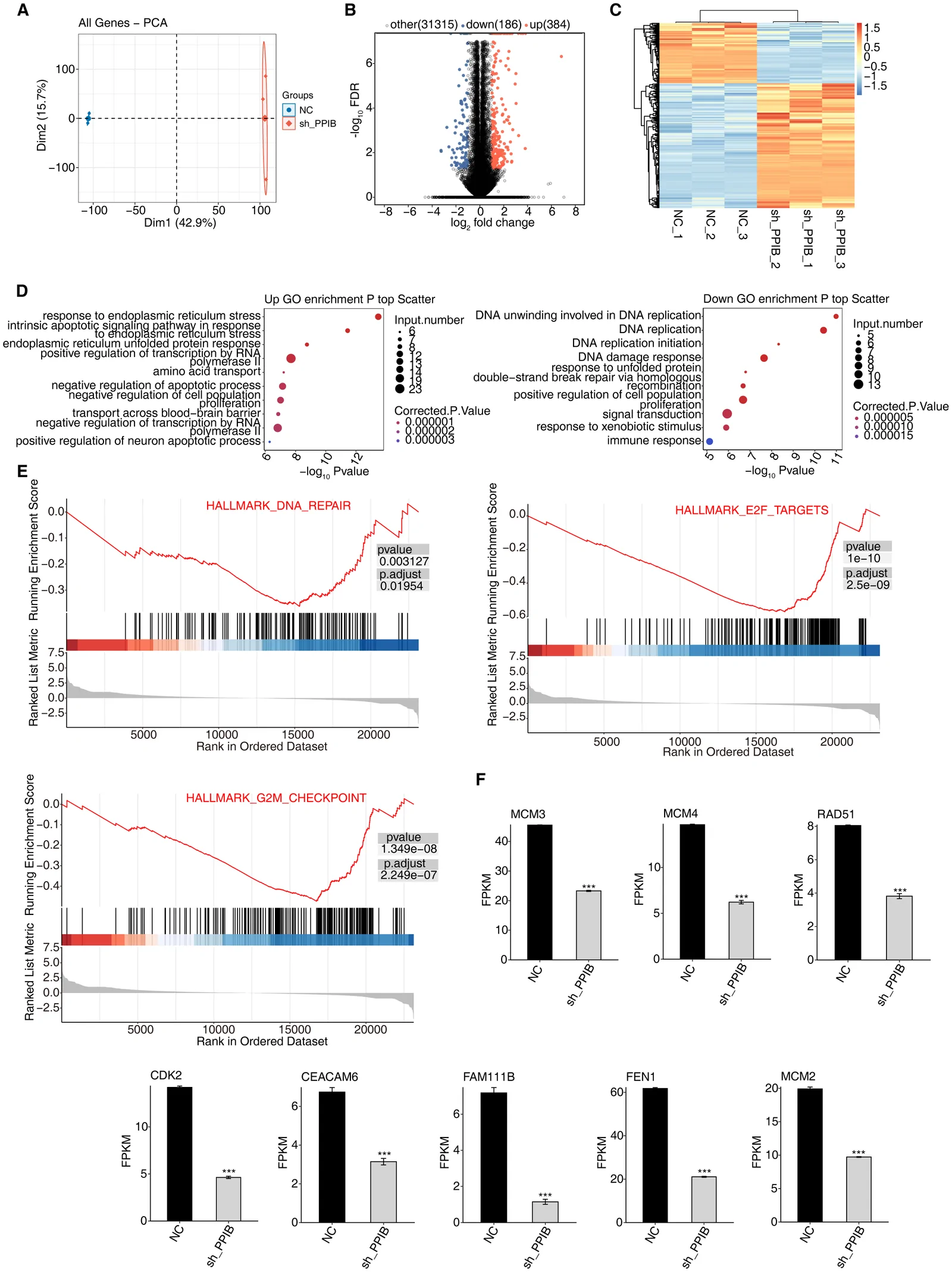
**(A)** Principal component analysis (PCA) based on FPKM values of all detected genes; ellipses indicate the confidence interval for each group. **(B)** Volcano plot of differentially expressed genes (DEGs) between sh-PPIB and sh-NC samples. **(C)** Heatmap showing the expression profile of DEGs. **(D)** GO biological process enrichment analysis of upregulated and downregulated DEGs. **(E)** GSEA plot showing pathway enrichment results. **(F)** Expression levels (FPKM) of representative downregulated genes. ***P < 0.001.

To characterize the transcriptional changes associated with *PPIB* silencing, we performed RNA sequencing in PC-3 cells. Principal component analysis (PCA) revealed robust clustering separation between the *PPIB* knockdown (sh-PPIB) and control (sh-NC) samples, reflecting substantial transcriptome-wide remodeling following *PPIB* silencing (**Figure 5A**). Differential expression analysis identified 570 significantly altered genes, including 384 upregulated and 186 downregulated genes (FDR < 0.05) (**Figure 5B**). Unsupervised hierarchical clustering further demonstrated good consistency within each group and clear separation between groups (**Figure 5C**).

Gene Ontology (GO) enrichment analysis showed that the upregulated genes were primarily associated with endoplasmic reticulum stress, unfolded protein response, transcriptional regulation, and amino acid transport. In contrast, downregulated genes were enriched in DNA replication, homologous recombination repair, cell-cycle regulation, and mitogenic signaling pathways (**Figure 5D**).

To further assess coordinated pathway-level changes, gene set enrichment analysis (GSEA) was performed using the ranked whole-transcriptome dataset. The results showed significant negative enrichment of the DNA repair, E2F targets, and G2/M checkpoint gene sets in the sh-PPIB group (**Figure 5E**) (14, 15). Consistently, several representative genes within these pathways, including *MCM2*, *MCM3*, *MCM4*, *RAD51*, *CDK2*, *FEN1*, *FAM111B*, and *CEACAM6*, were downregulated following *PPIB* knockdown (**Figure 5F**).

qRT-PCR was additionally performed on 10 representative DEGs. Nine genes reproduced the direction observed by RNA-seq: RAD51, MCM2, CEACAM6, GINS1, CDK2, FEN1, and FAM111B were significantly downregulated, whereas PHGDH and SHMT2 were significantly upregulated after PPIB knockdown. MYO6 did not show a statistically significant change (**Supplementary Figure 3**).

### *PPIB* knockdown alters alternative splicing in PC-3 cells

**Figure 6:**
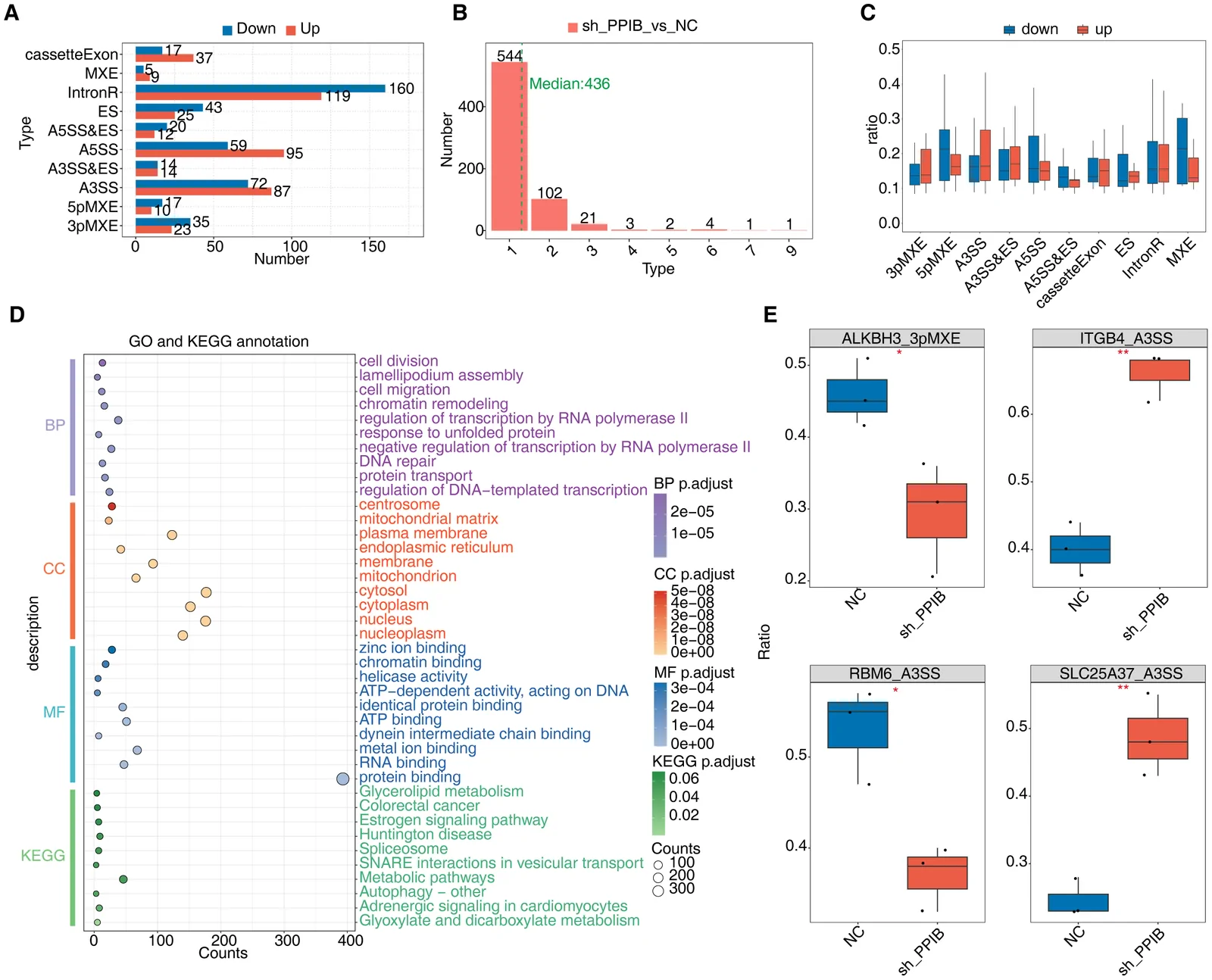
PPIB knockdown is associated with global remodeling of alternative splicing in PC-3 cells. **(A)** Distribution of regulated alternative splicing events (RASEs) across major splicing types. **(B)** Number of genes affected by RASEs (|Diff| > 0.15, FDR < 0.05). **(C)** Comparison of splicing ratios between upregulated and downregulated ASEs in representative event types. **(D)** Functional enrichment analysis of ASE-associated genes. **(E)** Representative candidate genes with altered splicing after PPIB knockdown. *P < 0.05, **P < 0.01.

*PPIB* depletion resulted in 873 significantly altered alternative splicing events (ASEs), among which intron retention (IR) was the most frequent type (**Figure 6A**). The majority of the affected genes exhibited only one significantly altered ASE (**Figure 6B**). In addition, the splicing ratios of downregulated ASEs were significantly higher than those of upregulated ASEs in both 5′-alternative mutually exclusive exon (5pMXE) and mutually exclusive exon (MXE) events (**Figure 6C**).

Functional enrichment analysis of ASE-associated genes showed significant enrichment in biological processes related to transcriptional regulation, protein transport, DNA damage response, unfolded protein response, chromatin organization, cell migration, and mitotic regulation. KEGG analysis further indicated enrichment in metabolic pathways, spliceosome, autophagy, SNARE-mediated vesicle transport, estrogen signaling, and glycerolipid metabolism (**Figure 6D**).

Among these ASE-associated genes, ALKBH3, ITGB4, RBM6, and SLC25A37 were selected as prostate cancer-related candidate genes based on significance and previous literature. Notably, ITGB4 was associated with cell migration-related splicing changes (**Figure 6E**). Event-specific qRT-PCR was used to evaluate five representative splicing events involving SHMT2, GINS1, RBM6, SLC25A37, and ALKBH3. The SHMT2 and SLC25A37 AS ratios increased, whereas the RBM6 and ALKBH3 AS ratios decreased, in the same direction as the RNA-seq estimates. In contrast, the GINS1 AS ratio increased by qRT-PCR, in the opposite direction predicted by RNA-seq. Thus, four of the five tested events showed directionally concordant changes, while the GINS1 event was not independently confirmed (**Supplementary Figure 4**; **Supplementary Table 2**).

### Integrated analysis links *PPIB* to metabolic pathway changes in prostate cancer

**Figure 7:**
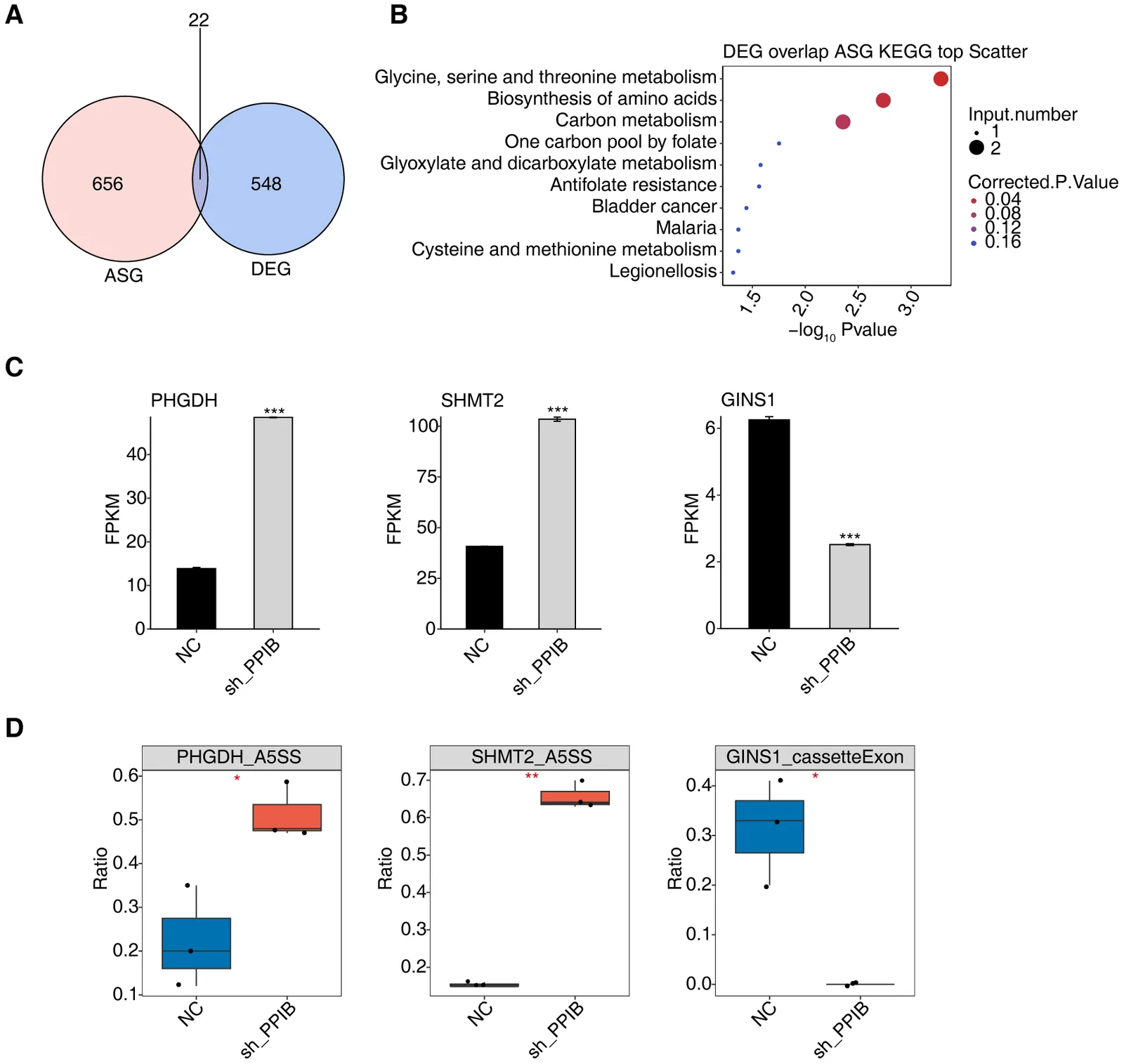
**(A)** Venn diagram showing the overlap between differentially expressed genes (DEGs) and alternative splicing-associated genes (ASGs). **(B)** KEGG enrichment analysis of overlapping genes. **(C)** Expression levels (FPKM) of representative genes with coordinated expression and splicing changes. **(D)** Splicing ratios of representative alternative splicing events. *P ≤ 0.05, **P ≤ 0.01, ***P ≤ 0.001.

A combined analysis integrating differentially expressed genes (DEGs) and genes exhibiting statistically significant alternative splicing events uncovered 22 genes concurrently dysregulated at both the transcriptional and post-transcriptional levels (**Figure 7A**). Enrichment mapping revealed strong overrepresentation of these co-dysregulated genes in key metabolic pathways—namely, folate-mediated one-carbon metabolism; glycine, serine, and threonine biosynthesis and degradation; amino acid synthesis; central carbon metabolism; glyoxylate and dicarboxylate metabolism; and mechanisms conferring antifolate resistance (**Figure 7B**).

Among these dual-altered genes, SHMT2, PHGDH, and GINS1 were selected as candidate genes for descriptive analysis because they showed coordinated expression and/or splicing changes and are relevant to cancer biology. Notably, SHMT2 and PHGDH were enriched in metabolism-related KEGG pathways. The expression levels (FPKM) and splicing ratios of these three genes are shown in **Figures 7C, D**. The splicing change of PHGDH shown in **Figure 7D** was derived from RNA-seq analysis and was not further validated by qRT-PCR; therefore, this event should be interpreted as a candidate PPIB-associated splicing event rather than definitive evidence of direct PHGDH splicing regulation.

## Discussion

TCGA-PRAD analysis showed significantly higher PPIB mRNA expression in prostate cancer. In the paired IHC cohort, tumor regions had higher H-scores in an exploratory ROI-level comparison, whereas the primary paired patient-level analysis showed a higher mean tumor H-score that did not reach statistical significance. This distinction is important because multiple ROIs from the same patient are not independent observations. In PC-3 cells—a model marked by PTEN deficiency and persistent PI3K/AKT/mTOR pathway activation (16)—silencing PPIB significantly impaired cellular proliferation, motility, and invasive capacity, implying that PPIB may contribute functionally to key oncogenic behaviors. PPIB depletion was also associated with a modest increase in apoptotic cells; however, because the absolute apoptosis rate was low, this observation should be interpreted cautiously and not overemphasized. Although PPIB knockdown was associated with reduced cell proliferation, migration, invasion, and xenograft tumor growth, the precise molecular pathways mediating these effects remain incompletely defined. In particular, the present study did not directly assess whether PPIB modulates key oncogenic signaling axes, such as the PI3K/AKT/mTOR cascade, or other established tumorigenic pathways. Therefore, the observed phenotypic changes should be interpreted as being associated with PPIB depletion, while the specific downstream signaling mechanisms remain to be determined.

The shRNA-resistant PPIB rescue experiments strengthened the specificity of the migration and invasion findings, as re-expression of PPIB significantly reversed both phenotypes caused by PPIB knockdown. Migratory activity returned to a level that was not detectably different from the control group, whereas invasive activity exceeded the control level. The latter finding may reflect supraphysiological PPIB expression rather than restoration to endogenous control levels. Accordingly, the rescue data support a PPIB-dependent contribution to these phenotypes, while the use of a single shRNA and a single prostate cancer cell line should still be considered.

Several methodological constraints should also be considered. First, the majority of functional experiments were performed in PC-3 cells, an androgen-independent prostate cancer model. These findings were not validated in additional prostate cancer cell lines, such as androgen-responsive LNCaP cells or another metastatic model such as DU145, which limits the generalizability to heterogeneous prostate cancer subtypes. Second, Annexin V/PI analysis showed only a modest increase in the absolute number of apoptotic cells. Supplementary immunoblotting showed higher levels of cleaved caspase-3, but no significant change in cleaved PARP1; thus, the biochemical evidence supporting apoptosis activation was insufficient, and apoptosis should not be presented as the principal mechanism. Third, N-cadherin decreased after PPIB knockdown, whereas E-cadherin and Vimentin were unchanged. This partial marker pattern does not establish a canonical EMT program. Finally, the observed changes in total SHMT2, GINS1, and PHGDH protein levels are supportive of expression-level remodeling but do not validate specific splice isoforms or prove that these proteins mediate the phenotypic effects. The additional immunoblot findings were obtained in cultured PC-3 cells. A complete, biologically replicated xenograft tissue panel with cleaved caspase-3, cleaved PARP1, and EMT markers was not available for inclusion in this revision; therefore, the xenograft findings should not be interpreted as evidence of apoptosis activation or EMT modulation *in vivo*.

RNA-seq analysis after *PPIB* silencing showed reduced expression of several genes involved in DNA replication, homologous recombination repair, and cell-cycle regulation. Representative genes included *MCM2*, *MCM3*, *MCM4*, *RAD51*, *FEN1*, and *CDK2*. Because these genes are associated with replication initiation, DNA repair, and cell-cycle progression, their concurrent downregulation may contribute to the marked reduction in proliferation observed in PPIB-silenced PC-3 cells. Previous studies have shown that several of these genes, especially members of the MCM family, are frequently upregulated in prostate cancer and are associated with adverse clinicopathological features (17). In this context, the reduced expression of replication- and repair-related genes after *PPIB* knockdown is consistent with the growth-inhibitory phenotype observed in our model.

Along with these replication- and repair-related changes, integrative analysis also highlighted metabolic regulators, including *PHGDH* and *SHMT2*, that are central to serine biosynthesis and one-carbon metabolism (18–20). Dysregulation of these pathways can impair nucleotide supply and redox homeostasis and may contribute to broader cellular stress responses after *PPIB* depletion (18, 21). Nonetheless, the precise functional consequences of this metabolic remodeling will require dedicated metabolic profiling and rescue experiments (e.g., genetic restoration of *PHGDH*/*SHMT2* or targeted metabolic supplementation).

Our findings further suggest that *PPIB* may be involved in alternative splicing regulation in prostate cancer. In addition to changes in overall gene expression, RNA-seq analysis revealed widespread splicing alterations in PC-3 cells after *PPIB* knockdown, with intron retention (IR) representing the most frequent event type. Because abnormal IR and broader splice-factor dysregulation have been linked to prostate cancer progression and cancer biology, this result supports the possibility that *PPIB* depletion affects post-transcriptional regulation in these cells (22–25). Among the genes showing PPIB-associated splicing changes, *ALKBH3*, *ITGB4*, *RBM6*, and *SLC25A37* were of particular interest because each has been linked to processes relevant to prostate cancer, including RNA regulation, cell adhesion and migration, and metabolic function (26–28). However, these associations should be interpreted with caution, as the functional consequences of the individual splicing events were not directly tested in the present study.

*PPIB* is classically known as a peptidyl-prolyl cis-trans isomerase involved in protein folding and maturation. However, previous studies have suggested that *PPIB* may also participate in RNA-related processes in addition to its role in proteostasis (7, 29, 30). This provides a possible explanation for the broad transcriptional and splicing changes observed after *PPIB* knockdown in our model. At the same time, our study did not include direct RNA–protein interaction assays, such as RIP-seq or CLIP. Therefore, although the observed expression and splicing changes were associated with *PPIB* depletion, we cannot determine whether they reflect direct RNA binding by *PPIB* or indirect downstream effects.

Integrated analysis showed that the downstream effects of PPIB depletion were mainly related to metabolic pathways and cell-cycle-associated programs. SHMT2, PHGDH, and GINS1 emerged as representative candidates altered at the expression and/or splicing levels. Supplementary immunoblotting showed higher total SHMT2 and PHGDH and lower total GINS1 after PPIB knockdown, directionally supporting the corresponding gene-expression changes. However, total protein abundance cannot resolve individual splice isoforms or establish causal mediation. Given the recognized importance of one-carbon and serine metabolism in prostate cancer and the emerging therapeutic relevance of PHGDH and SHMT2, these findings support an association between PPIB loss and metabolic remodeling (18–21, 31), while the underlying mechanisms remain to be clarified.

GINS1 was included as a candidate gene because it showed coordinated transcriptomic and splicing alterations and is linked to DNA replication-related programs. However, its direct contribution to PPIB-mediated phenotypes was not functionally tested in this study; therefore, GINS1 should be interpreted as a candidate downstream factor rather than a validated mechanistic mediator.

Although PHGDH was highlighted because of its relevance to serine biosynthesis and prostate cancer metabolism, the PHGDH splicing event identified by RNA-seq was not independently validated by event-specific qRT-PCR or an isoform-specific protein assay. The increase in total PHGDH protein shown in **Supplementary Figure 6** does not validate the specific splicing event. Therefore, PHGDH should be considered a candidate downstream event associated with PPIB depletion rather than a confirmed direct splicing target of PPIB.

## Limitations

This study has several limitations. First, the majority of functional experiments were conducted using PC-3 cells, which may limit the generalizability of the findings. Validation in additional prostate cancer models would strengthen the conclusions. Second, although PPIB knockdown was associated with broad changes in gene expression and alternative splicing, we did not perform direct RNA–protein interaction assays. Therefore, the observed splicing changes should be interpreted as being associated with PPIB loss rather than as evidence of direct regulation. Third, \ clinical validation was limited, and larger, better-annotated cohorts will be needed to clarify the clinical relevance of PPIB in prostate cancer. Fourth, qRT-PCR normalization was based on a single endogenous reference gene, GAPDH, without formal stability validation under the present experimental conditions. This may introduce normalization bias; therefore, qRT-PCR findings should be interpreted as supportive rather than as standalone evidence. The main conclusions are supported by complementary RNA-seq, Western blotting, immunohistochemistry, xenograft, and functional assay data. Fifth, the IHC cohort included only 15 paired cases. The ROI-level comparison was exploratory because multiple ROIs from the same patient are correlated; the primary, paired patient-level analysis was based on ROI-averaged values and did not reach statistical significance. Although the available clinicopathological characteristics are summarized in **Table 1**, the cohort was not powered for clinicopathological correlation analyses. In addition, although digital image analysis outputs were reviewed by pathology personnel, formal blinding was not documented. Finally, the PHGDH splicing event was identified in the RNA-seq data, but it was not validated by event-specific qRT-PCR or isoform-specific protein analysis. The total PHGDH Western blot cannot distinguish splice isoforms, limiting our ability to draw firm conclusions regarding PHGDH splicing regulation.

Although re-expression of shRNA-resistant PPIB reversed the migration and invasion phenotypes, the functional experiments still relied on one PPIB-targeting shRNA and one prostate cancer cell line. In addition, PPIB expression in the rescue group exceeded that of the control cells, and invasion in the rescue group surpassed the control level. Validation using an independent shRNA, additional prostate cancer models, and an expression-matched rescue system would further strengthen the specificity and generalizability of these findings.

The supplementary immunoblot analyses were limited to one cell line and three biological replicates per group. The apoptosis-associated markers were not fully concordant, and the EMT markers did not form a canonical pattern. In addition, SHMT2, GINS1, and PHGDH were measured as total proteins without isoform-specific resolution or downstream functional rescue. These protein data should therefore be interpreted as supportive rather than definitive mechanistic validation, and GINS1 remains a candidate requiring further functional investigation. In addition, the apoptosis- and EMT-related immunoblot analyses were performed in cultured PC-3 cells rather than xenograft tissues. Because a complete, biologically replicated xenograft tissue panel was not available, the *in vivo* data support reduced tumor growth but do not establish apoptosis activation or EMT modulation *in vivo*.

## Conclusions

Collectively, these results suggest that PPIB may contribute to prostate cancer progression and is associated with broad changes in gene expression and alternative splicing. In PC-3 cells, PPIB knockdown was associated with reduced proliferation, migration, invasion, and xenograft tumor growth. Transcriptomic profiling further indicated enrichment of differentially expressed genes in biological processes related to DNA repair, cell-cycle regulation, and metabolism-related pathways. Re-expression of shRNA-resistant PPIB reversed the migration and invasion defects associated with PPIB knockdown, strengthening the link between PPIB depletion and these phenotypes. Together, the phenotypic and molecular findings provide a foundation for future mechanistic studies, including validation in additional prostate cancer models, use of an independent shRNA and an expression-matched rescue system, and targeted functional analysis of candidate downstream mediators.

## Data Availability

The raw RNA-sequencing data presented in this study have been deposited in the Genome Sequence Archive for Human (GSA-Human) at the National Genomics Data Center, China National Center for Bioinformation, under accession number HRA020694.
