# Supplementary material for: PPIB and its role in prostate cancer: associations with transcriptomic and splicing alterations
Source: Front Oncol. 2026 Sep 8;16:1864404. doi: 10.3389/fonc.2026.1864404 (PMC13593429; doi:10.3389/fonc.2026.1864404)
Supplement: Supplementary file 1 [file SupplementaryFile1.docx]

**Supporting Information**

***PPIB and Its Role in Prostate Cancer: Associations with Transcriptomic and Splicing Alterations***

**Supplementary Figure S1. Annexin V/PI apoptosis analysis**

Flow cytometric apoptosis analysis of PC-3 cells after PPIB knockdown. Total Annexin V-positive cells showed a modest increase in sh-PPIB-275 compared with sh-NC (two-sided unpaired t test, P < 0.05; n = 3 biological replicates). Given the low absolute apoptosis rate, this result should be interpreted cautiously. Additional apoptosis-associated immunoblots are shown in Supplementary Figure S6.

**Supplementary Figure S2. Full-length western blots corresponding to Figure 2C**

Full-length PPIB and β-actin western blot images corresponding to Figure 2C. Lane groups are sh-PPIB-183, sh-NC, and sh-PPIB-275, with three lanes per group.

**Supplementary Figure S3. qRT-PCR validation of representative DEGs**

**Supplementary Figure S3 (continued)**

qRT-PCR validation plots for ten representative DEGs. Three independent biological replicates per group were measured in technical triplicate. The displayed points represent technical qRT-PCR measurements; biological-replicate-level summaries were used for manuscript interpretation. NC, sh-NC; SH, sh-PPIB-275.

**Supplementary Figure S4. Event-specific qRT-PCR validation of selected AS events**

Event-specific qRT-PCR validation plots for five alternative splicing events. Three independent biological replicates per group were measured in technical triplicate. GINS1 changed in the direction opposite to the RNA-seq estimate. Biological-replicate-level results are summarized in Supplementary Table S2. NC, sh-NC; SH, sh-PPIB-275.

Supplementary Figure S5. Validation of the shRNA-resistant PPIB rescue model

Validation of the shRNA-resistant PPIB rescue model in PC-3 cells. (A) qRT-PCR analysis of PPIB expression in sh-NC + OE-NC, sh-PPIB-275 + OE-NC, and sh-PPIB-275 + OE-PPIB cells. (B) Western blot detection of endogenous PPIB and FLAG-tagged ectopic PPIB, with β-actin as the loading control. The high PPIB expression in the rescue group represents supraphysiological re-expression. ns, not significant; ****P < 0.0001.

Supplementary Figure S6. Protein-level analysis after PPIB knockdown

Immunoblot analysis of selected apoptosis-associated proteins, EMT markers, and candidate downstream proteins after PPIB knockdown. (A) Representative blots for cleaved caspase-3, cleaved PARP1, N-cadherin, E-cadherin, Vimentin, SHMT2, GINS1, and PHGDH; β-actin served as the loading control. (B) Densitometric quantification normalized to β-actin. Cleaved caspase-3, SHMT2, and PHGDH increased; N-cadherin and GINS1 decreased; cleaved PARP1, E-cadherin, and Vimentin were not significantly changed. Data are presented as mean ± SD (n = 3 biological replicates per group). Two-sided unpaired t tests; *P < 0.05, **P < 0.01; ns, not significant. The marker pattern should not be interpreted as a complete canonical EMT response, and total-protein changes do not validate specific alternative-splicing events.

**Supplementary Table S1. Primer sequences used for qRT-PCR validation**

| **Application** | **Target / event** | **Primer name** | **Sequence (5′–3′)** |
| --- | --- | --- | --- |
| Reference gene | GAPDH | hum-GAPDH-F | GGTCGGAGTCAACGGATTTG |
| Reference gene | GAPDH | hum-GAPDH-R | GGAAGATGGTGATGGGATTTC |
| DEG qRT-PCR | RAD51 | RAD51-F | TGGTGTAGCAGTGGTAATCAC |
| DEG qRT-PCR | RAD51 | RAD51-R | GGGCGATGATATTTCCTCCAAT |
| DEG qRT-PCR | MCM2 | MCM2-F | GCCCGCTACCTTTCATTCC |
| DEG qRT-PCR | MCM2 | MCM2-R | TTATCCACCAAGTCCTTCTCAG |
| DEG qRT-PCR | CEACAM6 | CEACAM6-F | GGTCTCTCTTTGGCTGGAATT |
| DEG qRT-PCR | CEACAM6 | CEACAM6-R | TTAGCACCACTGGCTCAATG |
| DEG qRT-PCR | PHGDH | PHGDH-F | TTACCAGGCTGTGGGCTTG |
| DEG qRT-PCR | PHGDH | PHGDH-R | AGCATTGCAGGGTCAGAGG |
| DEG qRT-PCR | GINS1 | GINS1-F | AGTTACACGGCAGGCTGAG |
| DEG qRT-PCR | GINS1 | GINS1-R | TCAATCAAGGTGCCCAACAG |
| DEG qRT-PCR | CDK2 | CDK2-F | ACGACCCTAACAAGCGGATT |
| DEG qRT-PCR | CDK2 | CDK2-R | GGCTTCAAGAAGGCTATCAGAG |
| DEG qRT-PCR | FEN1 | FEN1-F | TGACCCACCTTGAGAGAG |
| DEG qRT-PCR | FEN1 | FEN1-R | GACACAGCCAGTAATCAGT |
| DEG qRT-PCR | SHMT2 | SHMT2-F | AGCCTTAACTTCTCGACAG |
| DEG qRT-PCR | SHMT2 | SHMT2-R | AGTCTTGCTCTTCACCTCT |
| DEG qRT-PCR | FAM111B | FAM111B-F | AGCAACATGGATGGAACTGAAG |
| DEG qRT-PCR | FAM111B | FAM111B-R | CACCCTCCACCCTCAAGTAG |
| DEG qRT-PCR | MYO6 | MYO6-F | GTGTGAGCTGAATCTTGAGGAG |
| DEG qRT-PCR | MYO6 | MYO6-R | TCGCATTCTGAAGGTACTGGAT |
| AS qRT-PCR | SHMT2 | SHMT2-M-F | AGGTGGAACGGCCTCTGCAGA |
| AS qRT-PCR | SHMT2 | SHMT2-AS-F | GCTTTTGAAGCCTCTGCAGA |
| AS qRT-PCR | SHMT2 | SHMT2-M+AS-R | GCCTGTCCTTCTCCCTCTGC |
| AS qRT-PCR | GINS1 | GINS1-M-F | TGCCTTCAACATGGAGTGGT |
| AS qRT-PCR | GINS1 | GINS1-AS-F | TAGCATACCTATGGAGTGGT |
| AS qRT-PCR | GINS1 | GINS1-M+AS-R | TTGGTGGTTTCATATCCTGT |
| AS qRT-PCR | RBM6 | RBM6-M+AS-F | AAAAGGATTTACTTGTTGGG |
| AS qRT-PCR | RBM6 | RBM6-AS-R | TTGGCTCCCACGAAAAGGTC |
| AS qRT-PCR | RBM6 | RBM6-M-R | ATTAGAGTTCCCGAAAAGGTC |
| AS qRT-PCR | SLC25A37 | SLC25A37-M+AS-F | AGGACTACGAGAACCTGCCGACTA |
| AS qRT-PCR | SLC25A37 | SLC25A37-AS-R | TCCACATTACCTTCACCGAGT |
| AS qRT-PCR | SLC25A37 | SLC25A37-M-R | GCATTCGTGTCTTCACCGAGT |
| AS qRT-PCR | ALKBH3 | ALKBH3-M+AS-F | TTTGTATCCTGGCTTTGTTG |
| AS qRT-PCR | ALKBH3 | ALKBH3-M-R | ACAGGGTGCCACCTCTCTGAT |
| AS qRT-PCR | ALKBH3 | ALKBH3-AS-R | CATTCTCTTCCCTCTCTGAT |

*Primer names and sequences are reproduced from the company-provided validation report. “M”, “AS”, and “M+AS” labels are retained as supplied. All sequences are shown in the 5′–3′ direction.*

**Supplementary Table S2. Event-specific qRT-PCR validation of selected alternative splicing events**

| **Gene / event** | **RNA-seq direction** | **sh-NC AS ratio** | **sh-PPIB AS ratio** | **qRT-PCR direction** | **P value** | **Concordance** |
| --- | --- | --- | --- | --- | --- | --- |
| SHMT2 | Up | 0.0047 ± 0.0003 | 0.1859 ± 0.0035 | Up | <0.0001 | Concordant |
| GINS1 | Down | 0.2750 ± 0.0107 | 0.3590 ± 0.0041 | Up | 0.0002 | Discordant |
| RBM6 | Down | 0.8761 ± 0.0045 | 0.8600 ± 0.0068 | Down | 0.0270 | Concordant |
| SLC25A37 | Up | 0.0959 ± 0.0027 | 0.1408 ± 0.0138 | Up | 0.0052 | Concordant |
| ALKBH3 | Down | 0.3477 ± 0.0270 | 0.2786 ± 0.0123 | Down | 0.0157 | Concordant |

*Data are mean ± SD from three independent biological replicates per group. Technical replicates were first summarized at the biological-sample level. P values were calculated using two-sided unpaired Student’s t tests on biological-replicate AS ratios. The GINS1 event changed in the opposite direction from the RNA-seq estimate.*
